# Rich at risk: socio-economic drivers of COVID-19 pandemic spread

**DOI:** 10.1186/s12948-020-00127-4

**Published:** 2020-07-01

**Authors:** Sebastiano Gangemi, Lucia Billeci, Alessandro Tonacci

**Affiliations:** 1 School and Operative Unit of Allergy and Clinical Immunology, Department of Clinical and Experimental Medicine, University Hospital “G. Martino”, Via Consolare Valeria SNC, 98125 Messina, Italy; 2 Institute of Clinical Physiology, National Research Council of Italy (IFC-CNR), Via Moruzzi 1, 56124 Pisa, Italy

**Keywords:** Coronavirus, COVID-19, Cytokine storm, Data analysis, Epidemiology, Pandemics, Respiratory distress

## Abstract

COVID-19, the novel coronavirus affecting the most part of worldwide countries since early 2020, is fast increasing its prevalence around the world, representing a significant emergency for the population and the health systems at large. While proper treatments are being developed, in-depth studies concerning its way of diffusion are necessary, in order to understand how the virus is actually spreading, through the investigation on some socio-economic indicators for the various countries in the world, retrieved through open-access data publicly available. The correlation analysis displayed significant relationships between COVID-19 incidence with several of such indicators, including the Gross Domestic Product per capita and the number of flights per capita, whereas mortality is mainly related to the main age of the population. All such data displayed an interesting mean to understand the way the virus has diffused worldwide, possibly representing the basis for future preventive measures to effectively challenge a new COVID-19 pandemic wave, but also other, similar pandemics.

## Main text

COVID-19, the novel coronavirus spreading worldwide since January 2020, accounts for more than 5.4 million cases in the world, with more than 340,000 deaths as of May, 26th, 2020 [1]. However, according to the report, albeit nearly any country in the world reported COVID-19 cases, noteworthy differences are present between continents as well as between single countries, with the largest amount of cases being reported in Americas and Europe, and a significantly lower prevalence observed in Africa, driving one to hypothesize either geographical or socio-economic factors driving such unbalance.

Indeed, with respect to the previous coronaviruses, including SARS-CoV and MERS-CoV, the new SARS-CoV-2 is spreading times faster and, as mentioned above, is affecting almost every country worldwide. To explain such different behavior of SARS-CoV-2 with respect to previous renowned coronaviruses, manifold reasons can be identified. Those include merely virological motivations, but also socio-economic arguments, such as population density, occurrence of social contacts, occupancy of confined spaces, countries’ economic statuses, and long-distance transportations [2].

However, albeit reasonable, and despite already partially hypothesized [2], to the best of our knowledge such relationship was not quantitatively assessed worldwide in the scientific literature up to now. As such, in order to fill in this gap, therefore to understand which of these discriminants could represent stronger drivers for the COVID-19 pandemic spread, we retrieved basic data on the open access website Wikipedia concerning socio-economic indicators of any country in the world, including their Population, Density, mean age, Gross Domestic Product (GDP) and Nominal GDP (both Per Capita), the Gini Coefficient of wealth distribution (an index of overall income inequality, ranging from 0, where everyone has the same income, to 1, where inequality is maximal), the Human Development Index (HDI, a composite index of life expectancy, education, and per capita income indicators ranging from 0 to 1). We also retrieved information about the fertility rate (Total Fertility Rate, TFR, defining the total number of children born or likely to be born to a woman in her life time) per each country according to the World Bank data [3] (https://data.worldbank.org/indicator/SP.DYN.TFRT.IN). Indicators about passenger traffic per country were added, retrieved from the last available data from the World Factbook of the Central Intelligence Agency [4]. Both classes of data were correlated with COVID-19-related data, including Cases and Deaths, both per million people, per each country, as retrieved on May, 27th, 2020 from Wikipedia [5]. Bivariate correlation was conducted using Pearson’s Correlation Index, corrected by Bonferroni post hoc analysis for multiple comparison.

As displayed in Table 1, the ratio of COVID-19 cases per million people was found to be significantly correlated with several socio-economic indicators, as with transportation-related factors. In particular, for COVID-19 cases, a moderate positive correlation was observed with the GDP Per Capita, the number of flights per Capita, the Nominal GDP Per Capita and the HDI. Beyond these findings, weak correlations were also seen with the mean population age of each country and the TFR (inverse relationship).

**Table 1:** Correlations between socio-economic and pandemic data (r: Pearson’s correlation index; *p < 0.005, **p < 0.001, according to the Pearson’s correlation index after Bonferroni’s post hoc for multiple comparisons)

	Population	Density	Age	GDP per capita	GDP nominal per capita	Gini	HDI	TFR	Flights	Flights per capita
Cases per 1 M										
r	− 0.053	0.114	0.371	0.632	0.481	− 0.200	0.408	− 0.328	0.211	0.520
p	0.479	0.132	< 0.001**	< 0.001**	< 0.001**	0.012	< 0.001**	< 0.001**	0.009	< 0.001**
Deaths per 1 M										
r	− 0.018	0.023	0.402	0.325	0.328	− 0.283	0.356	− 0.277	0.200	0.190
p	0.823	0.770	< 0.001**	< 0.001**	< 0.001**	0.001*	< 0.001**	< 0.001**	0.002*	0.023

Similar results were found when correlating the ratio of COVID-19 deaths per million people with the same socio-economic indicators mentioned above. In this second analysis, more indirect relationships had to be expected, since the number of deaths can be also affected by other factors, including the ratio of elderly people, or the capacity of each national health system to successfully cure a wide number of patients. Indeed, this analysis displayed correlation indices that are lower than those presented before, albeit remaining significant in most cases. In particular, for COVID-19 deaths, positive correlations were seen with age, expectedly being the most largely correlated among the indicators studied, the HDI, the GDP Nominal Per Capita, GDP Per Capita, TFR, Gini Coefficient (inverse relationship for the latter two), and the overall number of flights, whereas the number of flights per capita were excluded from the significance due to the Bonferroni post hoc correction. The negative correlation with Gini Coefficient could be explained by the fact that in countries with high levels of social disparity, little chances of social integration are present, thus leading to a lower spread of the virus.

Interestingly, data concerning the overall population and related density were not correlated with the number of COVID-19 cases per million inhabitants in world’s countries, nor with the number of deaths per million.

Taken together, these results lead to the understanding that, aside important clinical studies that should be performed to more clearly understanding the mechanisms of action of spread and the therapies more suitable for effectively tailoring the COVID-19 pandemic, several socio-economic drivers should be considered when studying the virus spread.

Indeed, in the era of globalization, with fast worldwide massive passenger transportations and continuous social contacts among people from all around the world, the speed and extensiveness of viruses’ propagation is times higher than just a few years ago, and countries with more frequent airplane connections with the rest of the world are more susceptible to this kind of occurrence [2]. At the same time, socio-economic indicators are also important determinants of pandemic spread, this fact possibly having manifold explanations, among which the higher number of social contacts (i.e., people living in countries with higher economic status are likely to attend a larger number of social events and to spend more time in overcrowded places, possibly paving the way for an easier virus diffusion) [6] and the higher efficiency of national health systems [7], that could affect the number of COVID-19 identified cases. Obviously, the aging of the population also makes countries at different risks for pandemic, with those with the older population featuring a higher amount of cases, on average, and the higher occurrence of deaths related to the COVID-19. However, interestingly, concerning the COVID-19 incidence, it appears that the mean age of the population is not the main factor influencing pandemic data, overtaken by the social and economic ratios mentioned above. On the other hand, higher population age is, otherwise, the first correlated indicator with mortality rate, making countries with the wider ratio of elderly people more at risk for COVID-19-related fatal occurrences.

Nonetheless, it is worth mentioning that a correlation itself does not necessarily imply consequentiality between two events that, in turn, should be studied in a more extensive manner and with the support of more complex statistical techniques, including Big Data Analytics and, eventually, taking advantage of Artificial Intelligence approaches.

However, despite such methodological limitations, this kind of correlations can lead to important considerations, potentially useful in a prospective framework. Indeed, the knowledge about the different countries’ susceptibility to this kind of viruses can allow drawing tailored preventive approaches based on such specificities to avoid, or reduce, future relapse in the COVID-19 pandemic or in future, somewhat similar conditions that might occur. For example, in case of a future, albeit not desirable, new pandemic outbreak or COVID-19 recurrence, measures like social distancing, smartworking or usage of Individual Protection Devices can be promptly adopted to quickly respond to the early emergency phases. Such preventive approaches will aim at making the population and the health systems ready to effectively face the related emergency and avoiding, at the same time, considerable loss of lives. In this way, the lesson learned worldwide due to the COVID-19 pandemic could be effectively employed for reducing the burden of future pandemic on economic, sanitary and social point of view.

## Data Availability

The datasets generated and/or analysed during the current study are available in the repositories mentioned within the references [1, 3–5].
